# SG-APSIC1054: Sputnik-V postvaccination immunologic responses in nasal mucosa: A prospective cohort study in Kazakhstan

**DOI:** 10.1017/ash.2023.13

**Published:** 2023-03-16

**Authors:** Irina Kadyrova, Svetlana Kolesnichenko, Ilya Korshukov, Yevgeniya Kolesnikova, Baurzhan Negmetzhanov, Yeldar Baiken, Aidana Sultanbekova, Sergey Yegorov, Dmitriy Babenko, Bakhyt Matkarimov, Gonzalo Hortelano, Matthew S. Miller

**Affiliations:** Karaganda Medical University, Karaganda, Kazakhstan; Karaganda Medical University, Karaganda, Kazakhstan; Karaganda Medical University, Karaganda, Kazakhstan; Karaganda Medical University, Karaganda, Kazakhstan; Nazarbayev University, Nur-Sultan, Kazakhstan; Nazarbayev University, Nur-Sultan, Kazakhstan; Karaganda Medical University, Karaganda, Kazakhstan; McMaster University, Hamilton, Ontario, Canada; Karaganda Medical University, Hemer, Germany; Nazarbayev University, Nur-Sultan, Kazakhstan; Nazarbayev University, Nur-Sultan, Kazakhstan; McMaster University, Hamilton, Ontario, Canada

## Abstract

**Objectives:** Sputnik-V (Gam-COVID-Vac) is a recombinant adenoviral (rAdv) vector-based, COVID-19 vaccine now used in >70 countries. Mucosal immunity is thought to be important for protection against COVID-19. We did a prospective cohort study to assess Sputnik-V–elicited mucosal SARS-CoV-2 antibody responses. **Methods:** We divided 82 COVID-19–free participants into prior COVID-19 and no prior COVID-19 groups and followed them at day 21 after Sputnik-V dose 1′ (rAd5) and dose 2′ (rAd26). Nasopharyngeal swabs and blood were collected to perform SARS-CoV-2 diagnostic and immunologic assays. SARS-CoV-2 spike-specific IgG and IgA ELISAs were performed on both nasal swabs and blood. SARS-CoV-2 real-time RT-PCR testing was performed to exclude infectious influencing. **Results:** Nasal S-IgG levels increased 25-fold after dose 1′ (*P* < .001) and remained high after dose 2 in all participants. Prior COVID-19 exposure was associated with both elevated baseline mucosal IgG and IgA and higher postvaccination IgG, but not IgA, boost. Nasal IgA levels increased 16.5-fold after dose 1′ (*P* < .001) and remained high after dose 2’ in all participants. Compared to dose 1′, Sputnik-V dose 2′ did not further increase either mucosal IgG levels (*P* = .626) or IgA levels (*P* = .609). **Conclusions:** A single dose of Sputnik-V boosted mucosal SARS-CoV-2 immunity. The effects of Sputnik-V dose 2′ on mucosal immunity were minimal. These findings indicate (1) that intramuscularly administered adenoviral vaccines enhance SARS-CoV-2 immunity via both systemic and mucosal routes and (2) that cost-effectiveness and the efficacy of Sputnik-V vaccination could be improved by adjusting the current prime-booster regimen and extending the 21-day interval between the doses. Trial registration: Registered on ClinicalTrials.gov (no. NCT04871841).

